# Prognostic value of isolated tumour cells in sentinel lymph nodes in early-stage breast cancer: a prospective study

**DOI:** 10.1038/s41416-018-0052-7

**Published:** 2018-04-24

**Authors:** Jenni S. Liikanen, Marjut H. Leidenius, Heikki Joensuu, Jaana H. Vironen, Tuomo J. Meretoja

**Affiliations:** 1 Comprehensive Cancer Center, Breast Surgery Unit, Helsinki University Hospital and University of Helsinki, P.O. Box 263, FIN-00029 HUS Helsinki, Finland; 2 Comprehensive Cancer Center, Helsinki University Hospital and University of Helsinki, P.O. Box 180, FIN-00029 HUS Helsinki, Finland; 3 Abdominal Center, Helsinki University Hospital and University of Helsinki, P.O. Box 340, FIN-00029 HUS Helsinki, Finland

**Keywords:** Breast cancer, Surgical oncology

## Abstract

**Background:**

The prognostic significance of isolated tumour cells (ITCs) in the sentinel nodes (SNs) is controversial in early breast cancer, and some centres have abandoned immunohistochemistry to detect ITCs.

**Methods:**

Patients with unilateral pT1N0 breast cancer, operated between February 2001 and August 2005 at a university hospital were included in this prospective, population-based cohort study. Survival of 936 patients with or without isolated tumour cells (ITC) in their SNs were compared with the log-rank test and Cox regression analysis.

**Results:**

Eight hundred sixty one (92.0%) patients were ITC-negative (pN0i−) and 75 (8.0%) ITC-positive (pN0i+). Patients with ITC-positive cancer received more frequently adjuvant systemic therapies than those with ITC-negative cancer. The median follow-up time was 9.5 years. Ten-year distant disease-free survival was 95.3% in the pN0i− group and 88.8% in the pN0i+ group (*P* = 0.013). ITCs were an independent prognostic factor in a Cox regression model (HR = 2.34, 95% CI 1.09–5.04; *P* = 0.029), together with tumour Ki-67 proliferation index and diameter. ITCs were associated with unfavourable overall survival (*P* = 0.005) and breast cancer-specific survival (*P* = 0.001).

**Conclusions:**

We conclude that presence of ITCs in the SNs is an adverse prognostic factor in early small node-negative breast cancer, and may be considered in the decision-making for adjuvant therapy.

## Introduction

The regional lymph node status has long been known to be an important prognostic factor in early breast cancer.^1^ Preoperative axillary ultrasound and a subsequent sentinel node biopsy (SNB) are currently the standard of care in the staging of clinically node-negative breast cancer. After harvesting, the sentinel nodes (SNs) are evaluated with immunohistochemical (IHC) staining of several tissue sections, which allows reasonably accurate assessment of both isolated tumour cells (ITCs) and micrometastases, whereas routine haematoxylin and eosin (H&E) staining often fails to detect them.^2^

In 2002, the American Joint Committee on Cancer (AJCC) TNM-classification (the 6th edition) defined and distinguished ITCs (pN0i+) from micrometastases (pN1mi).^3^ Since the definition of the ITCs their clinical importance has been debated, some arguing ITCs to have true metastatic potential and prognostic importance, while others consider them as artefacts from benign transportation after tumour manipulation, or otherwise of little importance in prognostication.^4–6^ In the beginning of the SN era, even the detection of small tumour deposits (including ITCs) in the lymph nodes led to axillary lymph node dissection (ALND), but data from clinical trials and other evidence now indicates that ALND does not improve survival of patients with limited SN involvement.^7–9^ Consequently, several guidelines now recommend to stop looking for ITCs, and many centres have abandoned IHC analysis of the SNs altogether.^9,10^

Although it is evident that patients with only ITCs in their SNs do not benefit from an ALND, the long-term prognostic significance of ITCs and their impact on the decision-making regarding the need of adjuvant therapy is unclear.^10–15^ To assess the clinical significance of ITCs, we aimed to investigate in the present study the long-term prognostic importance of ITCs in a large population-based cohort of patients with early node-negative breast cancer.

## Patients and methods

### Study population

A total of 1865 patients who had not been treated with neoadjuvant systemic therapy underwent breast surgery for unilateral, invasive pT1 (the largest tumour diameter ≤ 2 cm) breast cancer at the Breast Surgery Unit of the Comprehensive Cancer Center of Helsinki University Hospital, Finland, between February 2001 and August 2005, and were considered for this prospective cohort study. Since the Breast Surgery Unit of the Helsinki University Hospital is the only dedicated breast unit in a defined geographical region encompassing the Helsinki metropolitan area, serving a population of approximately 1.5 million, and operates close to 100% of all breast cancer patients in this region, the current data are close of being population-based.

**Fig. 1:**
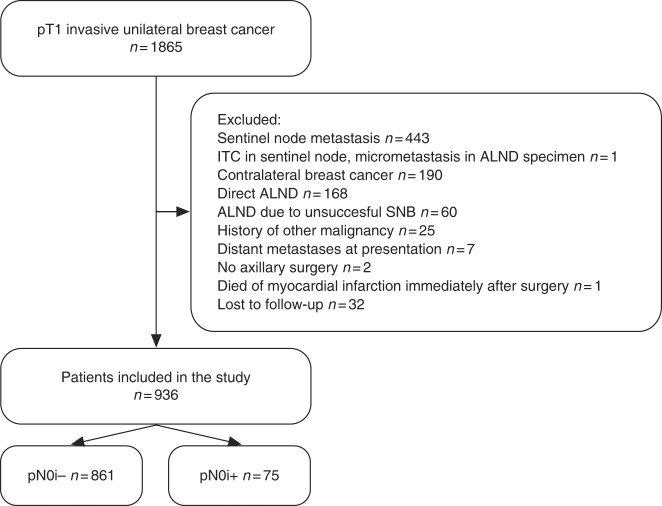
Patient inclusion flowchart

We excluded 929 (49.8%) patients from the cohort of 1865 patients for the reasons listed in Fig. 1, the most frequent reasons being presence of SN micrometastasis or macrometastasis (*n* = 443), presence of bilateral breast cancer or history of contralateral breast cancer (*n* = 190), ALND done without an SNB (*n* = 168), or technically unsuccessful SNB (*n* = 60). Patients with ITCs in the SNs after a SNB and with a micrometastasis or a macrometastasis in the non-sentinel nodes after an ALND were also excluded from the study population (*n* = *1*). The patients who had an ALND without a SN metastasis were excluded, since lymph nodes removed at ALND are generally not as meticulously investigated as the SNs, and ITCs are often missed. The remaining 936 patients with unilateral pN0 cancer form the present study population. The patients were categorised into two groups according to the SN biopsy results, either ITC-negative (pN0i−; *n* = 861 [92.0%]) or ITC-positive (pN0i+; *n* = 75 [8.0%]). The patient and tumour characteristics are provided in Table 1.

**Table 1:** Patient and tumour characteristics stratified by the presence of ITCs in the sentinel lymph nodes

	Number of pN0i− patients (%) (*n* *=* 861)	Number of pN0i+ patients (%) (*n* *=* 75)	*P*-value
Age at diagnosis (years)			
Median	58	57	0.262
Range	27–92	35–87	
Tumour size (mm)			
Median	12	13	0.021
Range	1–20	1–20	
Biopsy method			
FNAC	439 (51.0)	37 (49.3)	0.788
CNB	374 (43.4)	33 (44.0)	
Surgical biopsy	42 (4.9)	5 (6.7)	
NA	6 (0.7)	0 (0.0)	
Tumour histology			
Ductal	546 (63.4)	50 (66.7)	0.519
Lobular	155 (18.0)	15 (20.0)	
Other	160(18.6)	10 (13.3)	
Histological grade			
I	340 (39.5)	25 (33.3)	0.262
II	359 (41.7)	39 (52.0)	
III	151 (17.5)	11 (14.7)	
NA	11 (1.3)	0 (0.0.)	
Multifocality			
Unifocal	769 (89.3)	64 (85.3)	0.291
Multifocal	92 (10.7)	11 (14.7)	
Oestrogen receptor			
Negative	96 (11.1)	8 (10.7)	0.888
Positive	761 (88.4)	67 (89.2)	
NA	4 (0.5)		
Progesterone receptor			
Negative	267 (31.0)	19 (25.3)	0.286
Positive	587 (68.2)	56 (74.7)	
NA	7 (0.8)		
Ki-67 (MIB-1)			
0–19%	539 (62.6)	48 (64.0)	0.746
20–30%	166 (19.3)	12 (16.0)	
>30%	143 (16.6)	14 (18.7)	
NA	13 (1.5)	1 (1.3)	
HER-2 (CISH)			
Negative	675 (78.4)	55 (73.3)	0.424
Positive	52 (6.0)	4 (5.3)	
NA	134 (15.6)	16 (21.3)	

*ITC* isolated tumour cell, *FNAC* fine needle aspiration cytology, *CNB* core needle biopsy, *HER-2 (CISH)* human epidermal growth factor receptor 2 (chromogenic in situ hybridisation) *NA* not available

The research protocol was approved by an Ethics Committee at the Helsinki University Hospital.

### Surgery

Preoperative lymphoscintigraphy preceded the SN biopsy, and the SNs were identified at surgery using a gamma probe and blue dye as described in detail elsewhere.^16^ Nineteen (2.2%) of the 861 patients with pN0i− underwent a level I-II ALND due to surgeon or patient preference, and the majority of patients with pN0i+ underwent a level I-II ALND (62 [82.7%] out of 75, Table 2).

**Table 2:** Treatments given

	Number of pN0i− patients (%) (*n* *=* 861)	Number of pN0i+ patients (%) (*n* *=* 75)	*P*-value
Breast surgery			
Mastectomy	144 (16.7)	24 (32)	<0.001
BCS	717 (83.3)	51 (68)	
Axillary surgery			
SNB	842 (97.8)	13 (17.3)	<0.001
SNB and ALND	19 (2.2)	62 (82.7)	
Radiotherapy			
No	150 (17.4)	20 (26.7)	0.040
Yes	710 (82.5)	54 (72.0)	
NA	1 (0.1)	1 (1.3)	
Endocrine therapy			
No	480 (55.7)	16 (21.3)	<0.001
Yes	377 (43.8)	58 (77.3)	
NA	4 (0.5)	1 (1.4)	
Chemotherapy			
No	747 (86.8)	53 (70.7)	<0.001
Yes	112 (13.0)	21 (28.0)	
NA	2 (0.2)	1 (1.3)	

*ITC* isolated tumour cell, *BCS* breast-conserving surgery, *SNB* sentinel node biopsy, *ALND* axillary lymph node dissection, *NA* not available

### Histopathology

The breast and lymph node tissue specimens were sent to the pathology laboratory separately as fresh unfixed specimens and examined histologically by specialised breast pathologists. SNs were sliced in multiple sections 1–1.5 mm apart. An intraoperative frozen section analysis, including rapid IHC staining of the multiple sections, was performed. Rest of the SN tissue was formalin fixed, paraffin embedded and haematoxylin and eosin (H&E) stained in two sections. If no metastases were found, or if ITCs or micrometastasis was found in the SN frozen sections, an IHC staining for cytokeratin was performed, in addition to routine H&E staining. IHC staining was not done when a 2 mm or larger metastasis was found in frozen section analysis. The lymph nodes from the axillary clearance specimen were examined after staining with H&E. The histopathological analyses were performed in one specialist pathology laboratory.^17^

### Radiation therapy and adjuvant systemic therapy

Radiotherapy and systemic adjuvant treatments were administered according to the institutional guidelines (Table 2). After breast-conserving surgery, postoperative whole-breast radiotherapy was generally given to a cumulative dose of 50 Gy in 25 fractions. Tangential whole breast radiation fields were used after breast-conserving surgery. The target volume usually included the lower axilla. Separate axillary or supraclavicular fields were not used when the postsurgical axillary status was either pN0i− or pN0i+. A booster dose of 10 to 16 Gy was given to premenopausal women to the breast tumour site. Radiotherapy was not administered after mastectomy in this patient population with pT1 node-negative disease.

Adjuvant systemic treatment was administered depending on the patient and disease characteristics. In general, adjuvant systemic therapy was administered to patients considered to have a moderate-to-high risk of recurrence. Presence of ITCs in the SNs was not considered a mandatory indication for systemic adjuvant therapy. Premenopausal women with oestrogen receptor (ER) or progesterone receptor (PR)-positive cancer were scheduled to receive tamoxifen for a time period of 5 years, and postmenopausal women usually received an aromatase inhibitor for 5 years. Patients <65 years of age with moderate-to-high risk HER2-positive cancer received adjuvant trastuzumab and chemotherapy after May 2005, and a few patients prior to this within the context of a clinical trial.^18^

### Follow-up

The median follow-up time was 9.5 (interquartile range [IQR] 2.1) years after the date of primary breast surgery. In the subset of patients with pN0i− and pN0i+ cancer the median follow-up time was 9.5 (IQR 2.0) years and 9.0 (IQR 1.7) years, respectively. Regular follow-up visits were planned at one, three, and 5 years after breast surgery, and were organised at the Helsinki University Hospital. These visits included physical examination, blood cell counts, blood chemistry, and bilateral mammography. Breast and axillary ultrasound, bone isotope scan, and computerised tomography were performed whenever considered necessary. If concern of breast cancer recurrence occurred, the patients had access to further visits. After the first 5 years, the follow-up visits took place at the local health care centres or at private health care providers, according to patient preference. When cancer recurred, the patients were referred to the Helsinki University Hospital for further examinations and treatment. The date of breast cancer recurrence, the cause of death, and the date of death were extracted from the hospital records. In addition, data about cancer survival were obtained from the Finnish Cancer Registry, which has a coverage exceeding 95% in the population. Thirty-two patients were lost follow-up.

### Statistical methods

Frequency tables were analysed with the chi-squared test, and continuous distributions were compared with the Mann-Whitney *U*-test. Distant disease-free survival was calculated from the date of breast surgery to the date of first occurrence of breast cancer metastases outside of the locoregional region, censoring patients alive without distant metastases on the date of the last patient contact or on the date of death. Locoregional recurrence-free survival time was calculated from the date of breast surgery to the date of first regional lymph node or ipsilateral breast cancer recurrence, censoring patients without locoregional recurrence on the date of last follow-up contact or on the date of death. Breast cancer-specific survival was calculated from the date of breast surgery to the date of death considered to result from breast cancer, censoring patients without such an event on the last date of contact or on the date of death from an intercurrent cause. Overall survival was calculated from the date of surgery to the date of death from any cause, censoring patients who were alive on the date of the last contact.

Life tables were analysed using the Kaplan-Meier method, and survival between groups was compared with the log-rank test. A Cox proportional hazards regression model was used to assess the independent influence of covariables on survival. Variables with a *P*-value less than 0.1 in the univariable survival analysis were entered into a multivariable backward stepwise Cox regression analysis. Two-sided *P*-values < 0.05 were considered statistically significant. The multivariable survival analyses were calculated both with and without taking into account the given surgical and adjuvant systemic treatments. The statistical analyses were done using an IBM® SPSS® Statistics Version 20 software (SPSS Inc., Chicago, IL).

## Results

### Association between presence of ITCs and clinicopathological characteristics

There was no statistically significant difference between the pN0i− groups and pN0i+ groups regarding patient age, tumour histology, histological grade, the Ki-67 proliferation index, or cancer steroid hormone receptor status. The patients with pN0i+ cancer had slightly larger median tumour diameter as compared to patients with pN0i− disease (13 vs 12 mm, respectively; *P* *=* 0.021). The patients with pN0i− cancer had a longer median follow-up time than patients with pN0i+ cancer (9.5 vs 9 years, respectively; *P* *=* 0.016).

### ITCs and treatments given

The patients with ITCs in the SNs were generally treated more extensively. Patients with pN0i+ cancer underwent more often mastectomy and had an ALND, and they were treated more frequently with systemic adjuvant therapies as compared to the patients with pN0i− disease (Table 2).

### Survival

**Fig. 2:**
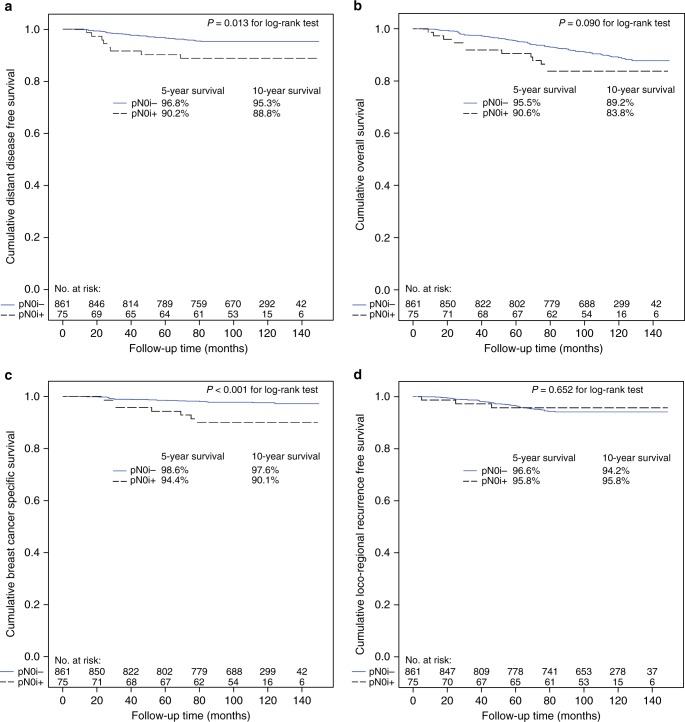
Distant disease-free survival (**a**), overall survival (**b**), breast cancer-specific survival (**c**), and locoregional recurrence-free survival (**d**) in the study cohort

Forty-seven (5.0%) patients were diagnosed with distant metastases during the follow-up, 39 (4.5%) out of the 861 patients in the pN0i− group, and 8 (10.7%) out of the 75 patients in the pN0i+ group. Ten-year distant disease-free survival was 95.3% in the pN0i− group and 88.8% in the pN0i+ group (univariable Cox regression hazard ratio [HR] 2.53, 95% CI 1.18–5.41; *P* = 0.017; Fig. 2a, Table 3). In a multivariable Cox proportional hazards regression analysis including all factors with prognostic influence the presence of ITCs was an independent prognostic factor for distant recurrence (HR 2.34, 95% CI 1.09–5.04; *P* *=* 0.029) together with tumour Ki-67 proliferation index and diameter (Table 3).

**Table 3:** Univariable and multivariable survival analyses for distant disease-free survival

	Univariable analysis			Multivariable analysis		
	HR	95% CI for HR	*P-*value	HR	95% CI for HR	*P*-value
Age (years)	1.00	0.98–1.03	0.960			
Tumour size (mm)	1.13	1.06–1.21	0.001	1.08	1.01–1.16	0.033
N0i− vs N0i+	2.53	1.18–5.41	0.017	2.34	1.09–5.04	0.029
Histological grade	2.48	1.66–3.70	<0.001	1.34	0.77–2.33	0.302
Tumour histology						
Ductal (reference)						
Lobular	0.55	0.21–1.40	0.208			
Other	1.25	0.63–2.50	0.518			
Tumour multifocality	1.18	0.50–2.79	0.699			
ER status	3.23	1.70–6.12	<0.001	1.04	0.43–2.49	0.934
PR status	2.20	1.24–3.89	0.007	1.51	0.82–2.77	0.183
Ki-67 (MIB-1)	2.37	1.71–3.30	<0.001	2.16	1.54–3.04	<0.001
HER-2 (CISH)	3.46	1.59–7.56	0.002	1.76	0.76–4.08	0.415

*FNAC* fine needle aspiration cytology, *CNB* core needle biopsy, *ER* oestrogen receptor, *PR* progesterone receptor, *HER-2 (CISH)* human epidermal growth factor receptor 2 (chromogenic in situ hybridisation). Age, Ki-67 and the histological grade were entered as continuous variables. Eighteen patients had one or more missing values in the final steps of the analyses

During the follow-up 104 (11.1%) patients died, 92 (10.7%) out of the 861 patients in the pN0i− group and 12 (16.0%) out of the 75 patients in the pN0i+ group. Ten-year overall survival was 89.2% in the pN0i− group and 83.8% in the pN0i+ group (univariable Cox regression HR 1.67, 95% CI 0.92–3.06; *P* = 0.094; Fig. 2b, Table 4). In a multivariable Cox regression analysis the presence of ITCs was an independent predictor for overall survival (HR 2.42, 95% CI 1.32–4.46; *P* *=* 0.005) together with age at diagnosis and the Ki-67 proliferation index (Table 4).

**Table 4:** Univariable and multivariable survival analyses for overall survival

	Univariable analysis			Multivariable analysis		
	HR	95% CI for HR	*P*-value	HR	95% CI for HR	*P*-value
Age (years)	1.09	1.07–1.11	<0.001	1.09	1.08–1.11	<0.001
Tumour size (mm)	1.06	1.01–1.11	0.011	1.03	0.98–1.08	0.299
N0i− vs N0i+	1.67	0.92–3.06	0.094	2.42	1.32–4.46	0.005
Histological grade	1.34	1.03–1.74	0.030	1.09	0.76–1.57	0.646
Tumour histology						
Ductal (reference)						
Lobular	0.94	0.56–1.58	0.803			
Other	0.84	0.49–1.45	0.538			
Tumour multifocality	0.93	0.48–1.78	0.814			
ER status	1.49	0.89–2.53	0.158			
PR status	0.94	0.61–1.45	0.777			
Ki-67 (MIB-1)	1.32	1.04–1.67	0.024	1.41	1.11–1.79	0.005
HER-2 (CISH)	0.42	0.13–1.34	0.142			

*FNAC* fine needle aspiration cytology, *CNB* core needle biopsy, *ER* oestrogen receptor, *PR* progesterone receptor, *HER-2 (CISH)* human epidermal growth factor receptor 2 (chromogenic in situ hybridisation). Age, Ki-67 and the histological grade were entered as continuous variables. Nineteen patients had one or more missing values in the final steps of the analyses

Only 27 (26%) of the 104 deaths were considered to have resulted from breast cancer (20 and 7 in the pN0i− and pN0i+ groups, respectively). The characteristics of patients with ITCs in the SNs and who died of breast cancer during the follow-up are provided in Supplementary Table 1. Ten-year breast cancer-specific survival was as high as 97.6% in the pN0i− group and 90.1% in the pN0i+ group (univariable Cox regression HR 4.29, 95% CI 1.81–10.15; *P* = 0.001; Fig. 2c). In a multivariable Cox proportional hazards regression analysis the presence of ITCs was an independent prognostic factor for breast cancer-specific survival (HR 4.13, 95% CI 1.75–9.77; *P* *=* 0.001) together with Ki-67 proliferation index (HR 2.93, 95% CI 1.86–4.60; *P* < 0.001).

A locoregional recurrence was diagnosed in 50 (5.3%) patients (47 [5.5%] and 3 [4.0%] in the pN0i− and pN0i+ groups, respectively). Ten-year locoregional recurrence-free survival was 94.2% in the pN0i− group and 95.8% in the pN0i+ group (log-rank *P* = 0.652; Fig. 2d).

The above survival analyses were performed without taking into account the treatment-related variables (the types of surgery and adjuvant treatments). The results remained largely unchanged with respect to the prognostic importance of ITCs in multivariable analyses for distant disease-free survival, overall survival, and breast cancer-specific survival, when the type of surgery and the adjuvant treatments given were included in the multivariable models (Supplementary Tables 2, 3, and 4). Supplementary information available at the British Journal of Cancer’s website.

## Discussion

We found that the presence of ITCs in the SNs was an independent predictor of distant recurrence in this cohort of patients with pT1 node-negative early breast cancer. The effect seemed consistent regardless of the survival end point selected, whether this was overall survival, breast cancer-specific survival, or distant disease-free survival, but the ITCs did not predict locoregional recurrence. Presence of ITCs was associated with inferior survival outcomes both in univariable and multivariable analyses, and regardless of whether the types of treatments administered were included in the multivariable models or not. Of note, the patients who had ITCs in the SNs had generally received more systemic adjuvant treatments than those who did not have ITCs, suggesting that the present estimates regarding the prognostic significance of the ITCs may be conservative. Patients who had no ITCs in the SNs had high 10-year distant disease-free and breast cancer-specific survival rates.

Previous studies have yielded controversial conclusions regarding the prognostic significance of ITCs in early breast cancer. While some studies have considered ITCs to have prognostic significance,^10,17,19–21^ others have not found such an association.^9,11–13,22–24^ The controversial results may, in part, be explained with the good short-term prognosis in pN0 breast cancer, and the relatively small number of survival events. However, as we found in the present and a previous study in this patient population,^17^ the number of survival events continues to increase with longer follow-up.^25^ Thus it seems likely that in order to draw firm conclusions on the prognostic importance of ITCs in the SNs long follow-up times are needed.

The results suggest strongly that finding ITCs in the SNs is associated with an increased risk of distant recurrence. The mechanisms of cancer cell seeding that lead to distant recurrence may be several, and the precise mechanisms may still be incompletely understood. Nonetheless, extensive surgical treatment of the axilla does not appear to result in survival benefits in patients with ITCs in the SNs.^9,26–29^ Rather, the presence of ITC might imply non-indolent biology of cancer, suggesting that besides the SNs, the tumour may have seeded micrometastases elsewhere in the body, which may sometimes manifest as overtly metastatic disease only after a long latency period. If this hypothesis is correct, aggressive local treatment of the axilla may not benefit the patient, whereas adjuvant systemic treatments might.

In node-negative breast cancer, the decision to administer adjuvant systemic therapy has traditionally depended on tumour characteristics such as size, grade, the proliferation index, the hormone receptor status, and the HER-2 status. More recently, biological subtypes, characterised with gene expression profiling, or approximated with surrogate IHC profiles, have been suggested as prognostic and predictive tools in the decision-making for adjuvant systemic therapy.^30^ One suggested criterion for adjuvant systemic therapy has been a more than 10% risk of recurrence during a 10-year follow-up. In the current study population, seven (9.3%) out of the 75 patients with pN0i+ cancer died of breast cancer, and eight (10.7%) others had distant metastases, adding up to a rate of 20% during a median follow-up time of 9.5 years. The MIRROR study demonstrated reduced survival associated with the presence on ITCs in the SNs in a series of 819 patients, where 513 patients with pN0i+ cancer did not receive systemic adjuvant therapy and 306 patients did. In this study systemic adjuvant therapy improved survival of patients with pN0i+ disease.^15^ Collectively, our data suggests that adjuvant systemic therapy might be considered for patients with pN0i+ cancer.

The main limitation of the study is that the analyses were based on a prospectively followed up cohort of patients, and not on a clinical trial population with previously defined inclusion and exclusion criteria and study procedures. The observational nature of the study makes it challenging to assess the effect of the given systemic therapies on prognosis. We limited the study to breast cancer patients with a small (pT1) primary tumour. A small proportion of the patients treated with a SNB have undetected metastases in the axilla.^31^ Axillary dissection was carried out substantially more frequently in the subset of patients with pN0i+ cancer than among those with pN0i− disease, and, therefore, undetected macroscopic axillary metastases, if any, were present more likely in the pN0i− group than in the pN0+ group, which may have reduced the observed survival difference between the groups.

Potential strengths of the study include a long follow-up time, the population-based setting, and a relatively large sample size, although the number of patients with ITCs in the SNs was relatively small. The SNs were analysed centrally using a standardised protocol, and the criteria for ITC detection remained unaltered during the study. In another study from our institute, the breast pathologists re-analysed the SNs histopathological findings from a part of the present patient population, and found only a very low rate of false-positive ITC findings.^32^ Most of the patients with ITC-positive cancer underwent a completion ALND, and patients with micrometastases or macrometastases in the non-sentinel nodes could be excluded from the study. Therefore, the ITC-positive patients in this study truly had pN0i+ cancer, a patient population that may currently be difficult to identify, since at present patients with ITC-positive cancer often do not undergo ALND.

## Conclusions

We conclude that the presence of ITCs in the SNs is associated with an increased risk of distant recurrence in patients with pT1N0M0 breast cancer. The current findings suggest that ITCs are a biological tumour characteristic that is independently associated with an increased risk for distant metastases, and may be considered when evaluating the need for systemic adjuvant therapy.

## Electronic supplementary material

Supplementary Table 1

Supplementary Table 2

Supplementary Table 3

Supplementary Table 4

## Availability of data and material

The original individual patient data is available from the authors as requested via e-mail.

## **Note**

This work is published under the standard licence to publish agreement. After 12 months the work will become freely available.
